# A novel approach to analyze the association characteristics between post-spliced introns and their corresponding mRNA

**DOI:** 10.3389/fgene.2023.1151172

**Published:** 2023-02-27

**Authors:** Suling Bo, Qiuying Sun, Pengfei Ning, Ningping Yuan, Yujie Weng, Ying Liang, Huitao Wang, Zhanyuan Lu, Zhongxian Li, Xiaoqing Zhao

**Affiliations:** ^1^ College of Computer Information, Inner Mongolia Medical University, Hohhot, China; ^2^ Department of Oncology, Inner Mongolia Cancer Hospital and The Affiliated People’s Hospital of Inner Mongolia Medical University, Hohhot, China; ^3^ Inner Mongolia Academy of Agricultural and Animal Husbandry Sciences, Hohhot, China; ^4^ School of Life Science, Inner Mongolia University, Hohhot, China; ^5^ Key Laboratory of Black Soil Protection And Utilization (Hohhot), Ministry of Agriculture and Rural Affairs, Hohhot, China; ^6^ Inner Mongolia Key Laboratory of Degradation Farmland Ecological Restoration and Pollution Control, Hohhot, China

**Keywords:** intron, alignment method, interaction, mRNA, evolutionary process

## Abstract

Studies have shown that post-spliced introns promote cell survival when nutrients are scarce, and intron loss/gain can influence many stages of mRNA metabolism. However, few approaches are currently available to study the correlation between intron sequences and their corresponding mature mRNA sequences. Here, based on the results of the improved Smith-Waterman local alignment-based algorithm method (SW method) and binding free energy weighted local alignment algorithm method (BFE method), the optimal matched segments between introns and their corresponding mature mRNAs in *Caenorhabditis elegans* (C.elegans) and their relative matching frequency (RF) distributions were obtained. The results showed that although the distributions of relative matching frequencies on mRNAs obtained by the BFE method were similar to the SW method, the interaction intensity in 5’and 3’untranslated regions (UTRs) regions was weaker than the SW method. The RF distributions in the exon-exon junction regions were comparable, the effects of long and short introns on mRNA and on the five functional sites with BFE method were similar to the SW method. However, the interaction intensity in 5’and 3’UTR regions with BFE method was weaker than with SW method. Although the matching rate and length distribution shape of the optimal matched fragment were consistent with the SW method, an increase in length was observed. The matching rates and the length of the optimal matched fragments were mainly in the range of 60%–80% and 20-30bp, respectively. Although we found that there were still matching preferences in the 5’and 3’UTR regions of the mRNAs with BFE, the matching intensities were significantly lower than the matching intensities between introns and their corresponding mRNAs with SW method. Overall, our findings suggest that the interaction between introns and mRNAs results from synergism among different types of sequences during the evolutionary process.

## 1 Introduction

The past decades have witnessed unprecedented medical breakthroughs. In this respect, the decade-long human genome project, ENCODE (Encyclopedia of DNA Elements) project improved our understanding that the human genome is a complex network system in which individual genes, regulatory elements, and DNA sequences unrelated to coding proteins interact in an overlapping manner to jointly control human physiological activities (The ENCODE Project Consortium, 2007; Zhang et al., 2007). The ENCODE project debunked the concept of “junk DNA”, which refers to very small protein-coding genes that are just one of many DNA elements with specific functions. It was also found that 93% of the DNA in the human genome could be transcribed into RNA, and many transcripts were non-coding RNA that could interact with each other (Comeron, 2001; Mattick and Gagen, 2001; Nott et al., 2003; Roy et al., 2003; Gabriel et al., 2005; Gazave et al., 2007).

Intron sequences represent an important and special class of ncRNA transcripts. They are transcribed together with mRNA and spliced to form a relatively independent class of ncRNA. The corresponding mature mRNA is the most important class of transcripts for storing genetic information and performing biological functions. According to the results of ENCODE project, an interaction is present between these two types of transcripts. Although it has been established that intron sequences (especially post-spliced introns) are regulatory elements with biological functions, their functions warrant further systematic study and exploration.

Intron sequences are carriers of important functional elements. It has been found that introns have many important biological functions and actively regulate gene expression. Six definite functions of spliceosome introns have been documented (Fedorova and Fedorov, 2003). Over the years, it has been shown that intron sequences are the vectors of important eukaryotic elements and play important biological functions in eukaryotic gene expression.

Intron loss/gain can affect many stages of mRNA metabolism. The gain and loss of intronic genes can affect the evolution of eukaryotes (Duret, 2001; Maquat and Carmichael, 2001; Jeffares et al., 2006; Nguyen et al., 2006; Roy and Hartl, 2006; Fawcett et al., 2011; Landen et al., 2022). Many experiments have found that introns play important roles in mRNA metabolism, such as transcription, splicing, nuclear transport and translation, as well as in regulating or maintaining the dynamic structure of mRNA (Le Hir et al., 2003; Elmonir et al., 2010; He et al., 2010; MariatiHo et al., 2010). At the transcription level, introns in many genes can significantly improve their transcription efficiency (Alexander et al., 2010; Akaike et al., 2011). In mice, the transcription levels of transgenes containing introns are 10–100-fold higher than those without introns (McKenzie and Brennan, 1996). It has been established that at the level of mRNA editing, introns are directly involved in splicing and contribute to synthesizing the 5’cap and 3’tail of the mRNA. An increasing body of evidence suggests that the cap structure can promote splicing and enhance the excision of its proximal first intron (Komarnitsky et al., 2000; Lewis and Izaurflde, 2004). During mRNA nuclear export, intron splicing is directly related to mRNA export (Le Hir et al., 2000; Gatfield et al., 2001; Kim et al., 2001; Lykke-Andersen et al., 2001). Early experiments have shown that mRNAs transcribed from cDNA cannot exit the nucleus and thus cannot express proteins, whereas mRNAs containing introns can exit the nucleus and express proteins (Ryu and Mertz, 1989; Rafiq et al., 1997). Besides, there is a growing consensus that introns can also affect the translation efficiency of mRNA (Torrado et al., 2009; Li and Pintel, 2012; Rocchi et al., 2012). Interestingly, Braddock et al. found that when a mature mRNA was injected directly into *Xenopus* oocytes, its translation was inhibited. This effect could be abolished by adding a spliceable intron to the 3’UTR of the gene or by injecting the FRGY2 antibody into the cytoplasm (Braddock et al., 1994). Indeed, intron deletion/gain can regulate gene expression at many stages of mRNA metabolism.

Introns can promote cell survival under stress. It is well-established that introns can regulate the survival and apoptosis of biological cells at the cellular level. In 2019, two research groups by Parenteau and Morgan found that yeast cells lack essential nutrients during the growth phase. Intriguingly, introns could accumulate by forming pre-mRNA (the Parenteau research group used pre-mRNA to judge the role of introns) or post-spliced intron (the Morgan research group used post-spliced) intron defines the function of introns) to adjust the rate of cell growth to adapt to this changing environment (Combs et al., 2006; Parenteau et al., 2008; Munding et al., 2013; Wanichthanarak et al., 2015; Awad et al., 2017; Venkataramanan et al., 2017; Wan et al., 2017; Morgan et al., 2019; Parenteau et al., 2019), thereby helping its survival. Results of these studies indicate that the huge family of intron sequences may have many potential functions and unknown binding ways, which warrant further exploration.

The use of binding free energy is an important means of studying RNA-RNA interactions. Based on the binding free energy principle, relative binding free energy calculation represents an effective means to study the interaction between biological macromolecules. During the analysis of the expression of coding RNA and the function of non-coding RNA, the minimum binding free energy method is used to predict its structure and further infer its close association, It has been established that 40%–70% of the known base pairs of RNA below 700bp can be correctly predicted (Deigan et al., 2009). The method of calculating free energy is also widely used in protein folding (Jackson, 1998; Schaefer et al., 1998; Selkoe, 2003), protein structure prediction (Bower et al., 1997; Zhang and Skolnick, 2005; Faraggi et al., 2009), molecular docking (Woo and Roux, 2005; Woo, 2008; Mitomo et al., 2009; Hay and Scrutton, 2012), and analysis of the interaction between biological macromolecules (Tollenaere, 1996; Anderson, 2003; Manke et al., 2003; Thomas et al., 2003; Gao et al., 2004; Martin and MacNeill, 2004; Prathipati et al., 2007). Introns and mRNAs are two types of RNA sequences. The binding free energy principle represents an important way to calculate the sequence interaction (mutual matching).

Based on the Smith-Waterman local alignment method, Li Hong et al. documented interactions between spliced introns and corresponding mRNA/CDS, and the distribution of their preferred interaction regions was universal among species. Since there are obvious differences in the binding free energies of base-base (A-T, C-G) during sequence matching, it is essential to fully consider these differences in binding free energies and to further study the matching association between introns and mRNA sequences from the perspective of thermodynamic stability.

Herein, the protein-coding genes in the genome of C. elegans were analyzed. The local high-throughput combined with free energy weighted local alignment method was used to perform local matching analysis of introns and mRNA sequences, to characterize the distribution of preferred regions of intron-associated fragments on mRNA sequences and near functional sites, and to analyze the sequence structure characteristics. We identified the putative biological functions of spliced introns and revealed the evolutionary relationship between introns and corresponding mRNA sequences, which lays the groundwork for exploring the potential biological functions of spliced introns and other ncRNAs.

## 2 Materials and methods

### 2.1 The gene sequences

The *C. elegans* genome and its annotation information were downloaded from the Beijing Multi Subnet of Gene Bank (ftp://ftp.cbi. pku.edu.cn/pub/database/genomes). The protein-coding genes of the *C. elegans* genome were selected as our dataset. In this dataset, the genes which contain ncRNAs and/or repetitive elements were excluded first. Next, the genes whose intron lengths are shorter than 40 bp were removed because the 5’splice region (about 8bp) and 3’splice region contain a pyrimidine-rich layer (about 30bp) of introns and functional regions conserved over evolutionary time (Petrov, 2002), and introns below 40 bp do not play other roles. Finally, after genes associated with alternative splicing were excluded, we obtained the *C. elegans* genome consisting of 5736 genes and 24312 introns.

### 2.2 Matched alignment

If interactions were found between introns and their mRNAs, there were positively matched segment pairs between introns and their mRNAs and *vice versa*. The potential interaction between introns and their mRNAs can be represented by the optimal matched segments (OMS). To obtain the OMS, the introns were first transformed into their complementary sequences. Next, the mRNAs were renamed as tested sequences and the complementary sequences of introns were renamed as aligned sequences; the assessment of similarity between different alignments was performed using an improved Smith-Waterman local alignment software (http://mobyle. pasteur. fr/cgi-bin/). Finally, the optimal similarity segments of the introns were transformed again into their complementary segments, which were the OMSs in the introns. During the similarity aligning process, the Ednafull matrix was used to calculate the OMS using the following parameters: 50.0 for the gap open penalty and 5.0 for the gap extension penalty.

**FIGURE 1:**
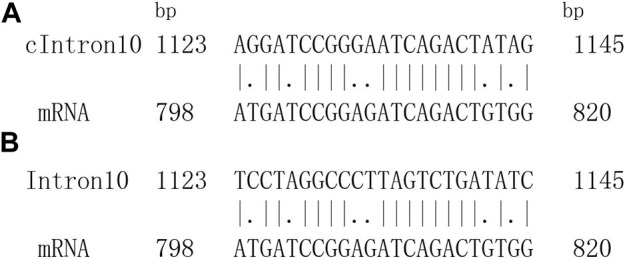
One optimal matching segment pair between introns sequence and corresponding mRNA sequence. **(A)** One optimal local alignment of fragment. cIntron10 represents complementary fragment of intron10. **(B)** Authentic matching alignment.

Accordingly, an objective optimal matched segment of a tested sequence and its aligned sequence could be obtained. The local alignment sketch map is shown in Figure 1.

The method based on the weighted comparison of binding free energy involves maximizing the number of hydrogen bonds and predicting the minimum free energy structure according to the negative correlation between the number of hydrogen bonds and the free energy (Zuker and Sankoff, 1984). The effect of base stacking force is not considered for the time being. Suppose the energy obtained by combining A-T/T-A base pair is EA-T/T-A, and the energy obtained by the G-C/C-G base pair is EG-C/C-G, then EA-T/T-A/EGC/CG ≈ 2:3. Due to the different release energy between A-T/T-A base pair and G-C/C-G base pair. In that case, different weights were assigned to them in the specific alignment process. The following principles were adopted during the matching process: If the base pair was correct, +3.0 would be awarded. +2.0 would be added if the base pair was A-T/T-A. If the base pair was G-C/C-G, it increased by +3.0. In this way, A correct matching of base pairs A-T/T-A yields +5.0 and a correct matching of base pairs G-C/C-G yields +6.0. If the base pairing was wrong, the penalty was −4.0. In this paper, the Ednafull matrix was still used to calculate The optimal matched fragments between the intron sequence and its corresponding mRNA sequence by using the binding free energy weighted local alignment method, and the selected parameters were as follows: The gap open penalty was −50.0 and the gap extension penalty was −5.0 for each base site. Finally, an optimal local matching fragment was obtained with the highest probability of interaction between the two sequences.

Definition 1 Sequence length normalization

Due to the different lengths of the tested sequences, they were normalized into 100 to obtain the relative site distributions using the following method.

The relative base site (k) of the *j*th base site in the tested sequence is

$$k=\left\{\begin{array}{l} \left[\left(\frac{100}{L}\right)*j\right]\;\left(\frac{100}{L}\right)*j\;\text{is}\;\text{integer}\\ \left[\left(\frac{100}{L}\right)*j\right]+1\;\left(\frac{100}{L}\right)*j\;\text{is}\;\text{non}-\text{integer} \end{array}\right.$$ (1)

Where, j means the *j*th base site of the tested sequence, L is the length of the tested sequence. The square brackets are gauss integer functions which mean to take integer part of a real number. Thus, the different lengths of the tested sequences were normalized to 100.

Definition 2 matched score function

For a tested sequence, the matched score function (fk) is

$$f_{k}=\left\{\begin{array}{l} 1\;k_{s}\leq k\leq k_{e}\\ 0\;k\pi k_{s}\;or\;k\phi k_{e} \end{array}\right.$$ (2)

Where, and represent the start base site and the end base site of the optimal matched segment in the normalized tested sequence. The effective value 1 is assigned to each base site within the optimal matched segment, while the ineffective value 0 is assigned to the base sites outside the optimal matched segment. Accordingly, the matched score values are assigned to each base site in the normalized tested sequences.

Definition 3 matched frequency

For the tested sequences, matched frequency function (F) is

$$F=\frac{1}{N}\overset{N}{\underset{i=1}{\sum }}f_{ik}$$ (3)

Where, i means the *i*th tested sequence, N means the number of the tested sequence. F reflects the interacting probability or the potential interaction intensity in the *k*th relative base site of the normalized tested sequences between the tested and aligned sequences.

Definition 4 average matched frequency

The average matched frequency function (<F>) for each base site is

$$\langle F\rangle =\frac{1}{N}\overset{N}{\underset{i=1}{\sum }}\frac{l_{i}}{L_{i}}$$ (4)

Where, li is the length of the optimal matched segment for the *i*th tested sequence. Li is the length of the *i*th tested sequence. For our normalized tested sequences, Li = 100. The <F> indicates the average matched frequency of the N-tested sequences, and it is a constant value for each tested set.

Definition 5 relative matched frequency

The relative matched frequency function (RF) of the *k*th base site in N tested sequence is

$$RF=\frac{F}{\langle F\rangle }$$ (5)

Where, RF reflects the relative bias of each base site in the N-tested sequences. If RF > 1, it indicates that the interaction in the *k*th base site is preferred, and the regions with RF > 1 are termed optimal matched regions (OMR). RF = 1 represents an average matched frequency of base sites for tested sequences.

### 2.3 Information entropy analysis

Information entropy can be used to characterize the organizational nature of a sequence. Second-order informational redundancy D2 is a suitable parameter to describe the adjacent base correlation of the sequence (Luo and Li, 1991; Li, 1990).

For an analyzed sequence, the second-order informational redundancy D2 is defined as:

D2=∑pijlog⁡2pijpipj≈12⁡ln⁡2∑pij−pipj2pipj (6)

Where pi or pj is the probability of the base i or j (i, j = A, C, G, U), and pij is the joint probability of the base pair ij in the sequence. A bigger D2 value means that the base correlation is stronger. For a finite sequence of length N, the fluctuation bound (f.b.) of D2 is D2 (f.b.) = 15.65/N (Luo and Li, 1991; Luo, 2004). When D2≥15.65/N, the neighboring bases occur not independently and the correlation does exist at 99% confidence level. Generally, D2≥0. For infinite random sequences, D2 = 0.

## 3 Results and discussion

### 3.1 Matched alignment between mature mRNAs and their introns

**FIGURE 2:**
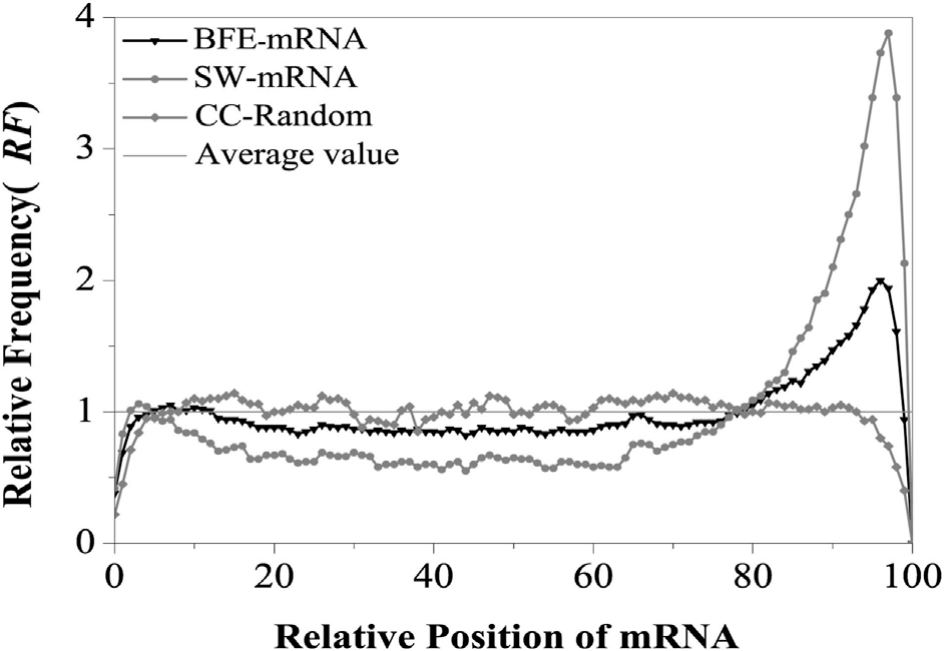
Relative Frequency (RF) distributions of mRNA. SW-mRNA means the RF value came from the base matching local alignment method. BFE-mRNA means the RF value came from the binding free energy weighted local alignment method. CC-Random means the local alignment were done between the component constraint random mRNA and their own component constraint random introns. Average value (RF = 1) means the theoretical average value of relative matched frequencies.

The relative matched frequency distribution (RF) on the mRNA sequence was assessed using the binding free energy weighted local alignment method and denoted as BFE-mRNA distribution. For the control, the relative matched frequency distribution on the mRNA sequence was assessed using the improved Smith-Waterman local alignment method and denoted as SW-mRNA distribution. The intron sequence was taken as the comparison sequence, and the corresponding mRNA sequence was taken as the test sequence. The optimal matched fragment between the two types of sequences was obtained using the binding free energy weighted local alignment method. Finally, the optimal matched frequency distribution on the BFE-mRNA sequence was obtained (Figure 2).

The results showed that the relative matched frequency (RF) distribution on the BFE-mRNA sequence was similar to the SW-mRNA sequence, and there were two preferred regions at the 5’and 3’ends of mRNAs (Appendix A:Supporting Information S1). The first region was located at about 5%–12% of the 5’end of the mRNA, and its peak value was about 1.05. The second region was located between 80% and 98% of the 3‘end of the mRNA, and its peak value was almost 2.0. The relative matched frequency of the 12%–80% region in the middle of the mRNA sequence was relatively low, slightly lower than the theoretical average, and its RF value fluctuated between 0.8 and 0.9. Compared with the CC-Random group (Appendix A:Supporting Information S2), The relative matched frequency (RF) of the BFE-mRNA sequence was more obvious in these two regions, and the difference in RF at the 3’end was highly significant (*t*-test, *p* < 0.00002).

Compared with SW-mRNA (Appendix A:Supporting Information S3), the preference of the relative matched frequency of the BFE-mRNA sequence was relatively weak at the 5’end region, exhibiting only one peak, which shifted slightly downstream. Although the distribution width of the preferred peak area at the 3’end remained unchanged, its peak value was only 1/2 that of the SW method and the difference was highly significant (*t*-test, *p* < 0.00003). The optimal relative matching frequency of the middle region was higher than the SW method, and it was significantly different from the CDS region (*t*-test, *p* < 0.00001) since the binding free energy weighted local alignment algorithm makes the optimal matched fragment combine with CDS with high G + C content.

The improved Smith-Waterman local alignment method and the binding free energy weighted local alignment method were used to describe the interaction between introns sequences and corresponding mRNA sequences. Analysis of the relative matched frequency distribution of mRNA sequence showed a consistent distribution preference by the two types of method. However, the regional difference in relative matched frequency distribution obtained by the base matched method was more obvious. To carefully analyze the distribution characteristics of each part of the mRNA sequence, The relative matched frequency distribution rule of each functional site region was studied next.

### 3.2 The distribution of relative matching frequency in functional site regions

There are many regions within the transcript that have regulatory functions, Such as translation initiation region, translation termination region and exon-exon junction region. The sequence of these functional domains plays a key role in the accurate expression of eukaryotic protein-coding genes. Therefore, it is necessary to explore the relative matched frequency of functional site regions.

The sites for translation initiation, translation termination and exon-exon junction are important functional regions of mRNA that regulate gene translation. Besides, the sequence of these functional regions is of great significance for the accurate expression of eukaryotic protein-coding genes. In this paper, we selected the ±60 bp regions of the translation start site (AUG), translation termination site (UAA) and exon-exon junction site (EE), which were denoted as AUG regions, UAA regions and EE regions, respectively, to analyze the relative matched frequency distribution of these regions by the BFE method, andcompared with that obtained by the SW method. (Castillo-Davis et al., 2002). Showed a close correlation between intron length and efficient gene expression. Halligan and Keightley et al. (Halligan and Keightley, 2006). Showed that long introns (>80bp) and short intron (≦80bp) distributions were significantly different. Therefore, we used 80bp as the threshold to distinguish between short and long introns.

Next, the introns were divided into three groups: An intron group, a long intron group and a short intron group named as intron, long intron and short intron, respectively. We compared and analyzed the overall differential characteristics of introns and the interactions between long and short introns with mRNA near functional sites. After obtaining The optimal matched fragment on the mRNA sequence, the distribution of the matched rate on the corresponding region was obtained by taking each functional site as the origin of the coordinate without length normalization.

#### 3.2.1 Relative matched frequency distribution of AUG and UAA regions

**FIGURE 3:**
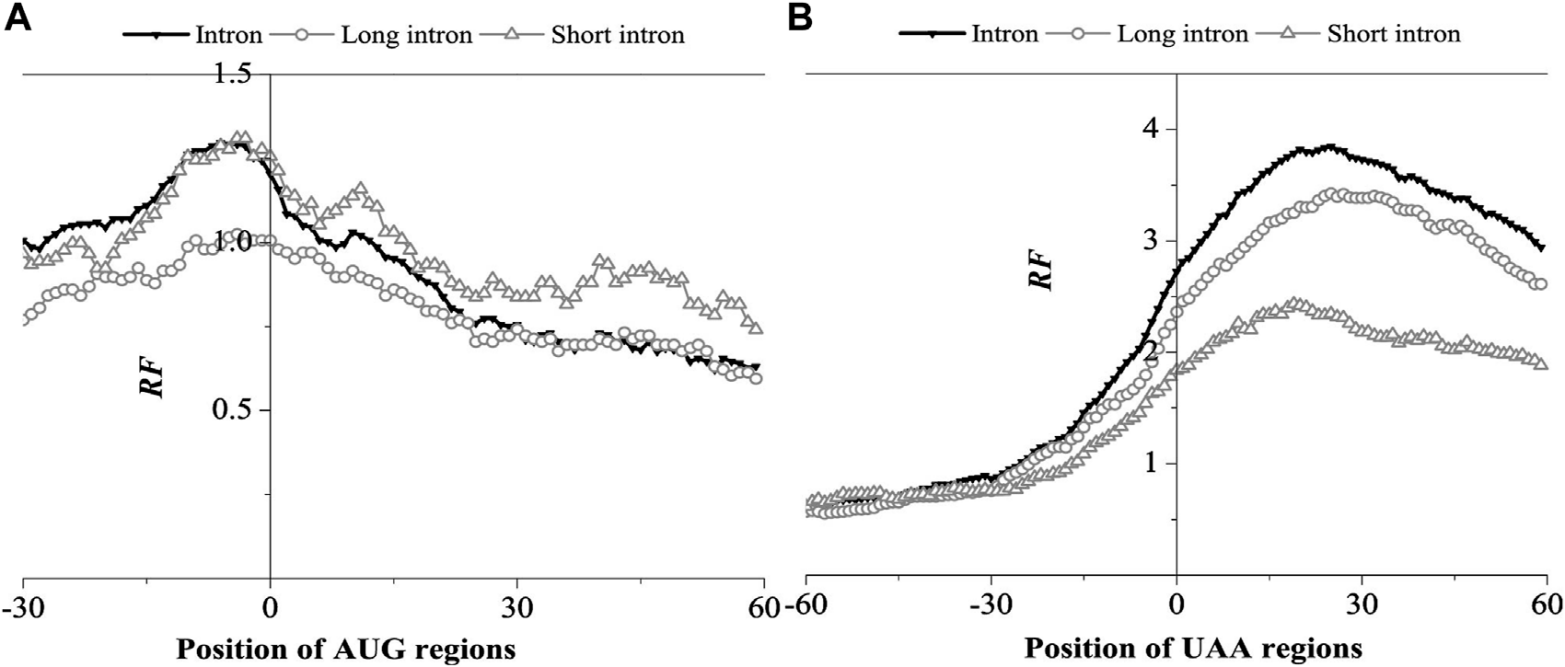
Relative Frequency (RF) distributions on AUG region **(A)** and UAA region **(B)** of mRNA. The RF distributions related long introns and short introns are also presented in the figure.

Analysis of the relative matched frequency distribution of translation initiation and termination regions was conducted to verify whether the matching preference region at both ends of the BFE-mRNA sequence is located in the UTR region. To avoid a boundary effect during comparison, mRNA sequences with 5‘UTR of less than 50bp and 3’UTR of less than 80bp were eliminated. Taking the first base of the translation start codon and translation stop codon as the coordinate origin, the relative matched frequency distribution characteristics of the translation start region and translation stop region were obtained (Figure 3).

As shown in Figure 3A, the peak distribution of mRNA was found at the 5’UTR and 3’UTR regions. The relative matched frequency at the 5’end gradually increased from −28bp of the AUG site and peaked at −10bp (RF = 1.3), then decreased to an average value of 10bp (RF = 1.0). Overall, The optimal matched fragments with introns ranged from −28bp to 10bp. The matched frequency of short introns in the AUG region was significantly higher than long introns, suggesting that short introns preferred interacting with the AUG region.

In the UAA region, the relative matched frequency distribution was significantly different from the AUG region (Figure 3B). From −28 bp of the UAA site, the relative matched frequency increased rapidly, the RF value reached 2.8 at the UAA site, peaked at about 28bp (RF = 3.8), and then gradually decreased, but the RF value remained high. In the 3’UTR region, it suggested that the interaction region is longer and much stronger than in the 5’UTR region. In addition, in the 3‘UTR region, the interaction intensity of long introns was significantly higher than short introns, which is opposite to the AUG region, suggesting that long introns preferred to interact with the UAA region.

**FIGURE 4:**
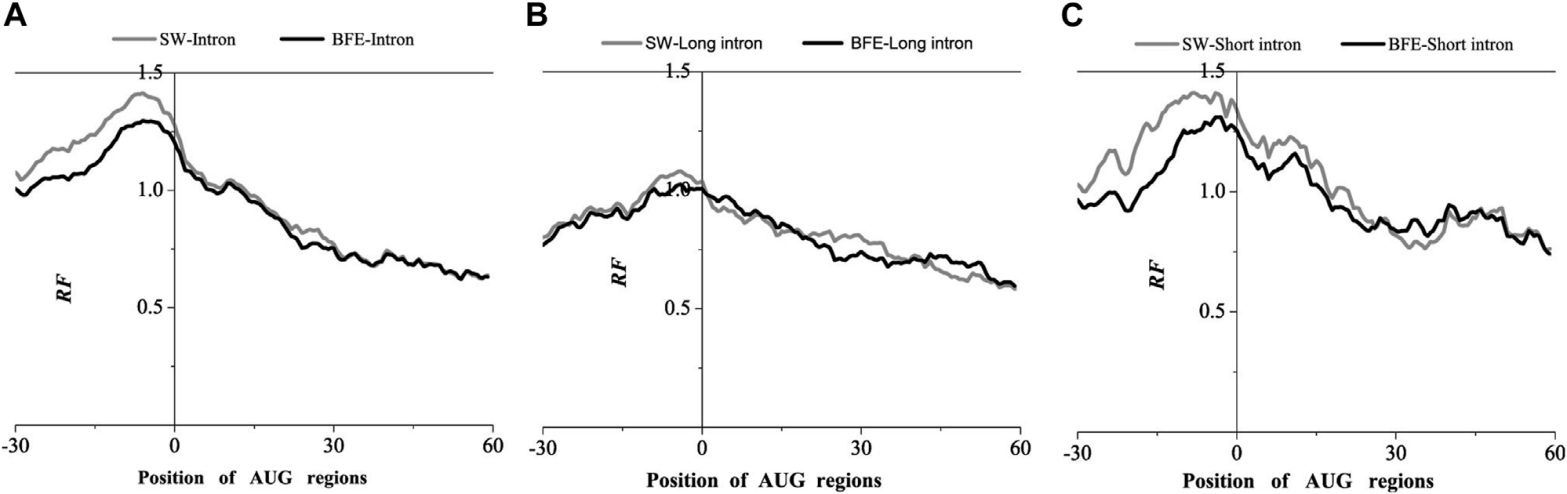
Comparations of Relative Frequency (RF) distributions between SW method and BFE method on AUG regions. **(A)** The total introns, **(B)** the long introns and **(C)** the short introns.

**FIGURE 5:**
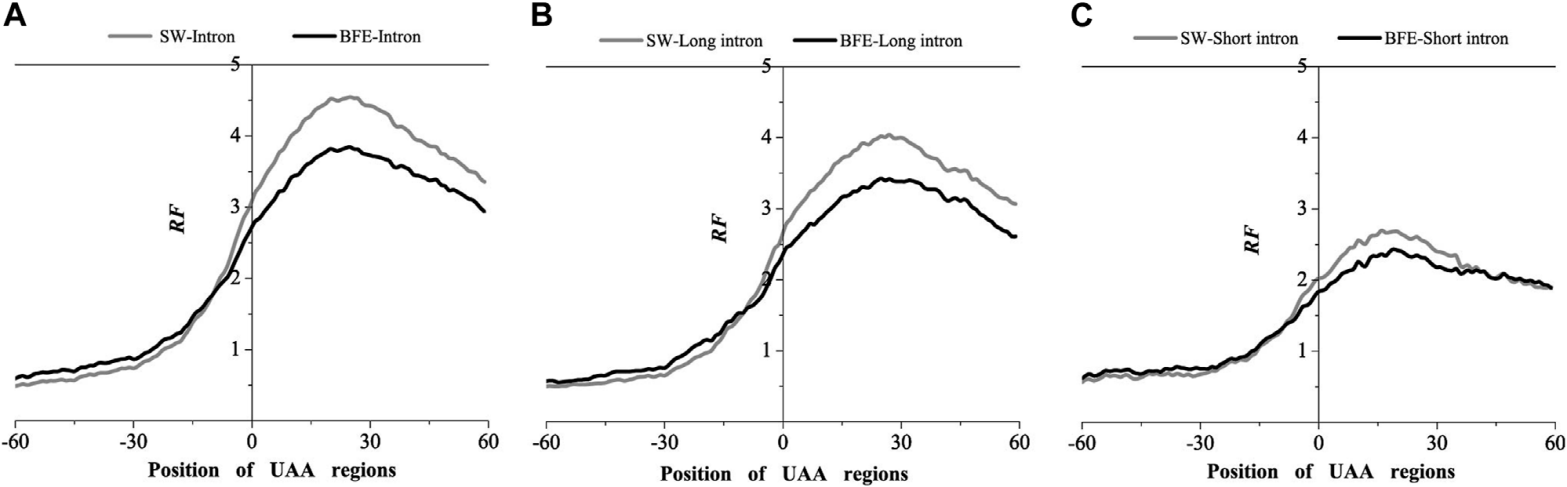
Comparations of Relative Frequency (RF) distributions between SW method and BFE method on UAA regions. **(A)** The total introns, **(B)** the long introns and **(C)** the short introns.

The results obtained by the base matching method (SW method) and the binding free energy weighted method (BFE method) were compared in the AUG and UAA region (Figures 4, 5).

Compared with the base matching method (SW method), the optimal matching frequency distribution trend of the AUG and UAA regions obtained by the binding free energy weighted methe binding free energy weighted local alignment method (BFE method) was similar. In the AUG region, The relative matched frequency distribution of both the whole intron group and the short intron group was slightly lower than that obtained by the base matching method (Figure 4), and the difference was more significant near the −10bp region of the AUG site. For long introns, the distribution was almost the same. In the UAA region, The relative matched frequency of the whole intron and long intron was significantly lower than the SW-mRNA group. Moreover, there was no significant difference in the distribution of short introns (Figure 5).

The analysis results of the two representative interactions indicated a significant preference for intron-mRNA interaction in the UTR region, especially in the 3’UTR region. Short and long introns preferentially acted in the 5’and 3’UTR region, respectively.

#### 3.2.2 Relative matched frequency distribution in EE region

**FIGURE 6:**
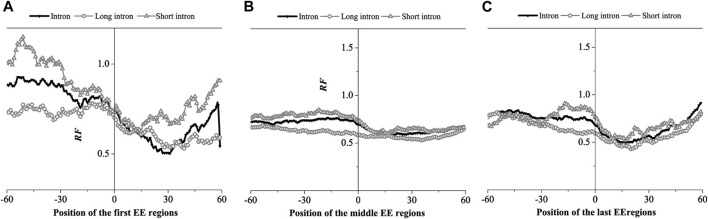
Relative Frequency (RF) distributions on the exon-exon junction (EE) regions of the mRNA. **(A)** The first EE regions, **(B)** the middle EE regions, **(C)** the last EE regions. The RF distributions related long introns and short introns are also presented in the figure.

The EE region is divided into three groups: The first exon connection region, the intermediate exon connection region and the last exon connection region, composed of the corresponding exon connection site ±60 bp region. The relative matched frequency distribution was obtained by the binding free energy weighted local alignment method (BFE method), as shown in Figure 6.

The relative matched frequency distribution of EE regions in the three groups was similar. The relative matched frequency of the upstream region of the exon-exon junction site was higher than the downstream region. The difference was more significant in the first and last exon regions and least significant in the middle exon region. The minimum values of the distributions occurred 30bp downstream of the first exon connection point, while it is about 15bp downstream of the last exon connection point. However, there was no obvious difference in the minimum values of the distributions at the middle exon-exon junction. It was also found that the relative matched frequency of short introns was higher than long introns in all three EE regions. Based on the findings of previous studies, we hypothesized that the region with low relative matched frequency might be the protein factor binding region.

**FIGURE 7:**
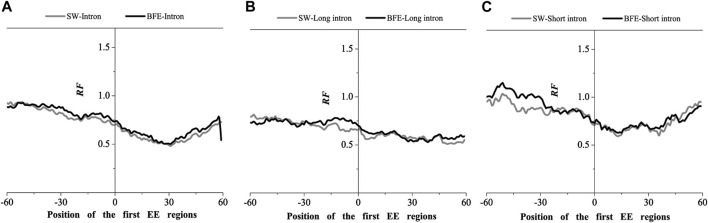
Comparations of Relative Frequency (RF) distributions between SW method and BFE method on the first EE regions. **(A)** the total introns, **(B)** the long introns,**(C)** the short introns.

**FIGURE 8:**
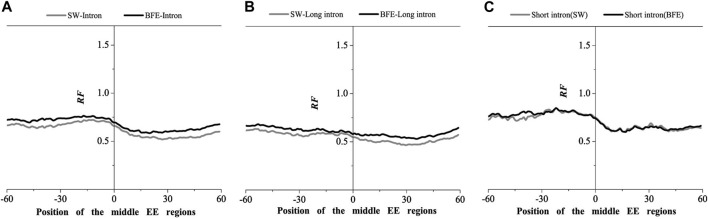
Comparations of Relative Frequency (RF) distributions between SW method and BFE method on the middle EE regions. **(A)** the total introns, **(B)** the long introns,**(C)** the short introns.

**FIGURE 9:**
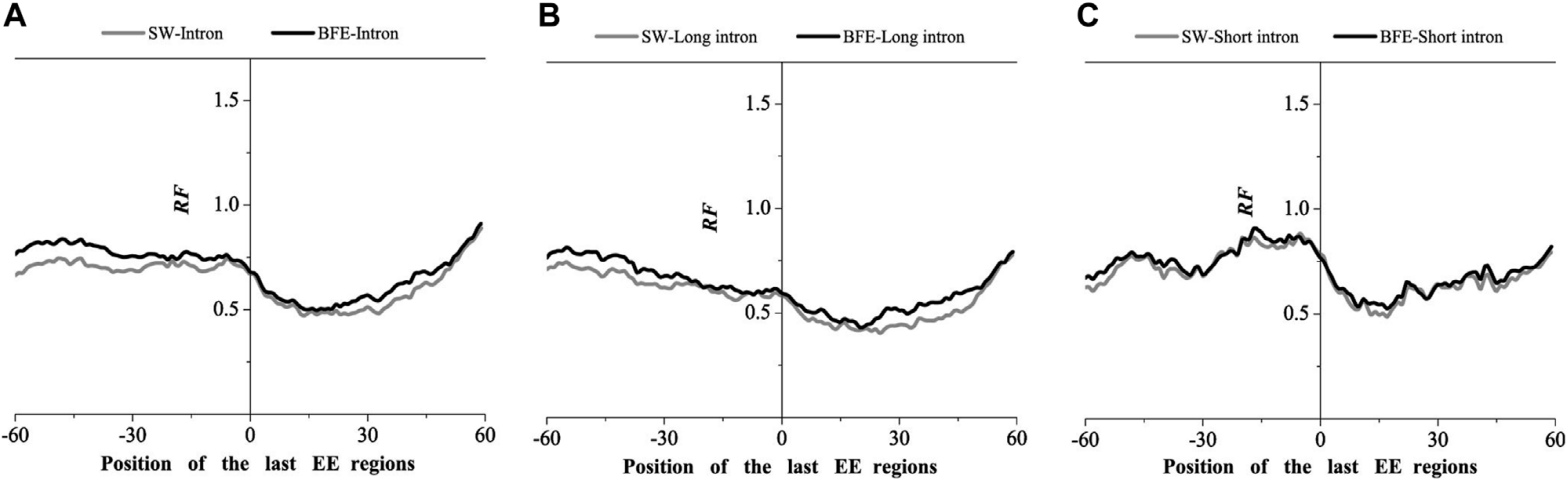
Comparations of Relative Frequency (RF) distributions between SW method and BFE method on the last EE regions. **(A)** the total introns, **(B)** the long introns, **(C)** the short introns.

We next compared the matched frequency distribution characteristics of the exon-exon junction regions of the mRNA group based on between the improved Smith-Waterman local alignment method and the binding free energy weighted local alignment method (BFE method). The mRNA group based on the improved Smith-Waterman local alignment method was used as the control group. The distribution of the optimal matched frequencies of the whole intron, long intron, and short intron groups on exon junction regions on mRNA based on the binding free energy weighted local alignment method was compared with that of the SW method group. The results were showed in Figures 7–9.

The optimal matched frequency distribution trend of the OMF in the junction region on the mRNA sequence (which is of the corresponding mRNA sequences and the intron sequences) based on the binding free energy weighted local alignment method was comparable to the SW method. In the exon-exon junction regions of the first, last and intermediate exons, although the weighted matched frequency distribution of the whole intron, long intron and short intron groups were slightly higher than the SW method (Figures 7–9), there was no significant difference between them.

These results indicated that the distribution of the matched frequency of exon junction regions obtained by SW method and BFE method is conservative. The matched frequency values of the exon-exon junction regions obtained by the BFE method were larger than those obtained by the SW method, which was caused by the tendency of the binding free energy weighted local alignment algorithm to combine the optimal matched fragment with CDS with high G + C content. The binding preference of intron sequence (especially short introns) and exon connection sites upstream regions suggests a preferred interaction between the intron sequence and the exon-exon junction region of the mRNA sequence. Besides, the process of short introns is more advantageous, which may be attributed to the fact that the biological function of short introns is mainly related to mRNA splicing or alternative splicing. These interesting results are worth thinking about.

### 3.3 Sequence characteristics of the optimal matched fragments

We calculated four sequence features of the optimal matched fragment pairs based on the BFE method, including the match rate distribution, length distribution, G + C content distribution and base association (D2 value). The results were compared with those obtained by the SW method.

#### 3.3.1 The distribution of match rate and length

**FIGURE 10:**
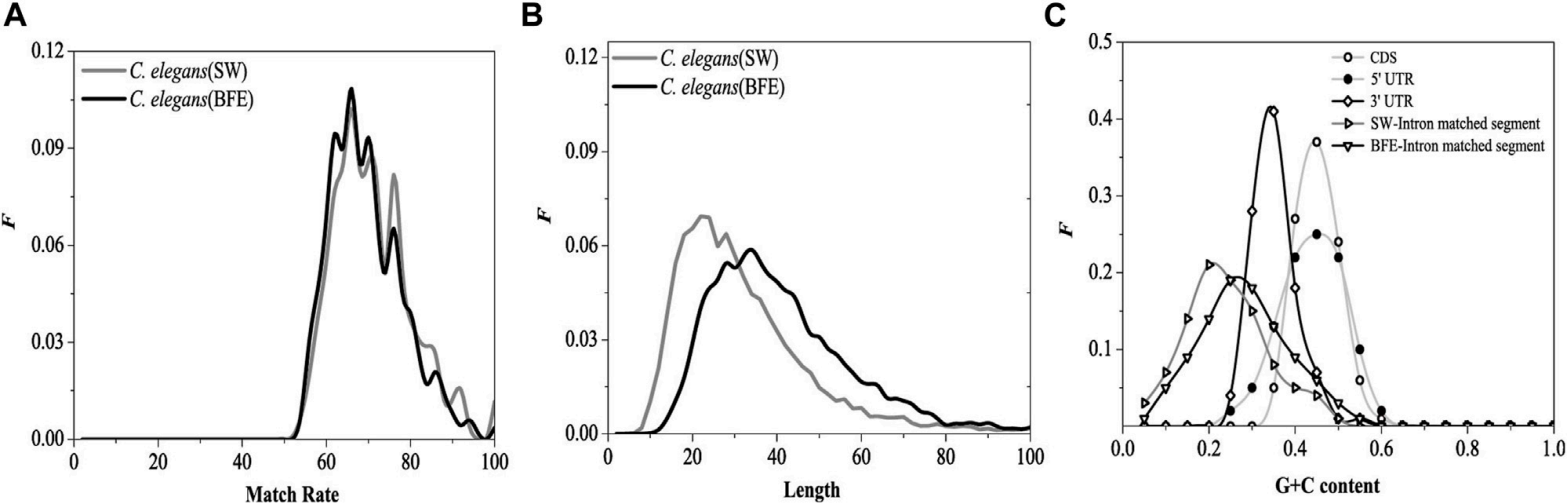
Distributions of the match rates **(A)**, the length **(B)** of optimal matched segments. SW represents the result came from SW method and BFE represents the result came from BFE method G + C content distributions **(C)** for CDS, 5, UTR, 3, UTR, optimal matched segments (SW method) and optimal matched segments (BFE method).

The match rate distribution of the optimal matched fragment of intron obtained by the BFE method is shown in Figure 10A. The distribution of the match rate of the optimal matched fragment obtained by the two methods was very similar, except that the distribution curves have relatively small fluctuations. The length distribution of the optimal matched fragment of intron obtained by the BFE method is shown in Figure 10B.

The functional fragments representing the interaction between introns and mRNA are a class of functional fragments similar to miRNA, and their match rate and the most length should be similar to miRNA fragments. The length of functional segments of siRNA was very conserved, ranging from 21 to 23 bp, while that of miRNA ranged from 18 to 25 bp. The most length of the optimal matching fragment by SW method was 23bp, and its characteristics were similar to miRNA fragments. However, the biologically roles of the interaction between introns and mRNA should be differ from the biologically roles of siRNA and miRNA, we believe that the biologically roles of the interaction between introns and mRNA should be protected mRNA from degradation and be beneficial to transport of mRNA from nucleus to cytoplasm. The interaction strength of between introns and mRNA should be weaker than siRNA and miRNA, and the lengths of the optimal matched segments (OMS) should be longer than siRNA and miRNA. Our results show that the maximum length obtained by BFE method is 36bp, which is quite different from miRNA fragment, and the mated rate obtained by BFE method is lower than SW method and siRNA and miRNA. So, the results by BFE method may have a biological significance.

#### 3.3.2 G + C content and D2 value

The G + C content distribution of the optimal matched fragment on the introns by the BFE method is shown in Figure 10C. The distribution range of G + C content in the optimal matched fragment of the BFE method was consistent with the SW method, but the peak region of G + C content was about 0.25, which moved toward high G + C content, it increased 0.05 compared with the SW method. The reason for the general increase in G + C content is caused by the fact that was the preference for intron fragments with high G + C content during selecting the optimal matched fragments by the binding free energy weighted local alignment method.

Their D2 values are calculated by formula (6), and the results are shown in Table 1. It can be found that the D2 value of the optimal matched fragment was significantly higher than CDS, 5 and 3’UTR sequences, it suggested the base association in the OMF was significantly stronger than the other three types of sequences, with a strong sequence structure. Besides, the D2 value of the optimal matched fragment by the BFE method was about 20% lower than the SW method, indicating that the former method can document the interaction between the intron and mRNA sequences and characterize their interaction.

**TABLE 1:** D 2 values of different sequences in *Caenorhabditis elegans* protein-coding genes.

	mRNA			Intron	
	CDS	5, UTR	3, UTR	OMS (SW)	OMS (BFE)
D 2	0.029	0.032	0.036	0.066	0.053

Note: OMS indicates The optimal matched fragment of the introns.

## 4 Conclusion

In the present study, the binding free energy weighted local alignment algorithm method was used to obtain the optimal matched fragment between the post-spliced intron and its corresponding mRNA sequence, and the relative matched frequency distribution on the mRNA and near the functional site. Our results showed that the relative matched frequency distribution obtained by the BFE method was similar to the SW method; there were the region of preference at the UTR region at both ends of the mRNA sequence was identified as a favorable region, especially in the 3’UTR region. However, the suggestion of the combination show that was more in favor of the optimal matched fragments with CDS with high G + C content, which was the weaker interaction in the 5’and 3’UTR regions, and higher in the middle CDS region than the SW method, when the BFE method was applied.

Moreover, we found that the region of preference of theshort introns in the 5’UTR region, and the long introns in the 3’UTR region, which consistent with the SW method. Besides, the relative matched frequency distribution in the exon connection region was similar to the SW method. The interaction intensity of the upstream connection point was greater than that of the downstream, and there was a minimal relative matching frequency distribution of the downstream of the first and last exon connection region, and the interaction of short introns was stronger than long intron sequences.

The match rate distribution and the length distribution shape of The optimal matched fragment were similar to the SW method, although an increase in optimal matching fragment length was observed. When the SW method was applied, the maximum value length was 23bp, and an increase to 36bp was observed with the BFE method. It was still broad (0.05–0.5) with the distribution range of the content of G + C of the optimal matched fragment, but the maximum value of the content of G + C by the SW and BFE methods was 0.2 and 0.25, respectively, which display the content of G + C by the BFE method was generally higher. Although the base correlation of the optimal matched fragment remained strong, it was slightly lower than the D2 value in the SW method. These results substantiate that the optimal matched fragment is a special sequence fragment with a highly structured organization.

Overall, the BFE method and SW method yielded similar results. However, it was the less intensity of the interaction between introns and corresponding mRNA by the BFE method, the length of the optimal matched fragements was longer, and the bases association or sequence structure of the OMF was relatively weaker. Compared with SW, the BFE method is more sensitive than the SW method for representations the RNA-RNA interaction and can avoid the false positives which may occur in SW method.

In conclusion, the BFE method and SW method yielded similar results, the results obtained by the BFE method and SW method were basically the same, indicated that the binding free energy weighted local alignment method can be used to predict the interaction between introns and their corresponding mRNAs. According to the comparison of the matched frequency distribution between introns and corresponding mRNA sequences, the BFE method was more conducive to predict the weak interaction between sequences with high G + C content. The sequence characteristics of the optimal matched fragments obtained by the BFE method implyed that the structures of sequence with longer length, higher G + C content and looser sequence structure are more likely to predict weak interactions between sequences with higher GC content, compared with those calculated by the SW method.

We advocate that using local base matching to characterize the interaction between introns and mRNAs has huge prospects.

## Data Availability

The original contributions presented in the study are included in the article/Supplementary Materials, further inquiries can be directed to the corresponding authors.

## Appendix A: Supplementary data to this article

1. Instruction about the database. txt (Taking an example).
2. Supporting Information S1-for the optimal matched regions located at UTR. txt.
3. Supporting Information S2-for CC-Random group. txt.
4. Supporting Information S3-for mRNA group. txt.
